# Association of psychosocial adversity and social information processing in children raised in a low-resource setting: an fNIRS study

**DOI:** 10.1016/j.dcn.2022.101125

**Published:** 2022-06-18

**Authors:** Laura Pirazzoli, Eileen Sullivan, Wanze Xie, John E. Richards, Chiara Bulgarelli, Sarah Lloyd-Fox, Talat Shama, Shahria H. Kakon, Rashidul Haque, William A. Jr. Petri, Charles A. Nelson

**Affiliations:** a Labs of Cognitive Neuroscience, Division of Developmental Medicine, Boston Children’s Hospital, Boston, MA, USA; b Harvard Medical School, Boston, MA, USA; c University of South Carolina, Columbia, SC, USA; d Icddrb, Dhaka, Bangladesh; e University of Virginia, Charlottesville, VA, USA; f Birkbeck, University of London, UK; g University of Cambridge, Cambridge, UK; h University College London, London, UK; i Harvard Graduate School of Education, Cambridge, MA, USA; j School of Psychological and Cognitive Sciences, Peking University, China; k PKU-IDG/McGovern Institute for Brain Research, Peking University, China

**Keywords:** fNIRS, Infancy, Low and middle income countries, Social cognition, Psychosocial adversity

## Abstract

Social cognition skills and socioemotional development are compromised in children growing up in low SES contexts, however, the mechanisms underlying this association remain unknown. Exposure to psychosocial risk factors early in life alters the child’s social milieu and in turn, could lead to atypical processing of social stimuli. In this study, we used functional Near Infrared Spectroscopy (fNIRS) to measure cortical responses to a social discrimination task in children raised in a low-resource setting at 6, 24, and 36 months. In addition, we assessed the relation between cortical responses to social and non-social information with psychosocial risk factors assessed using the Childhood Psychosocial Adversity Scale (CPAS). In line with previous findings, we observed specialization to social stimuli in cortical regions in all age groups. In addition, we found that risk factors were associated with social discrimination at 24 months (intimate partner violence and verbal abuse and family conflict) and 36 months (verbal abuse and family conflict and maternal depression) but not at 6 months. Overall, the results show that exposure to psychosocial adversity has more impact on social information processing in toddlerhood than earlier in infancy

## Highlights

• Social selectivity was measured with fNIRS in 6, 24 and 36 months Bangladeshi children.
• Psychosocial adversity impacts this measure of social information processing.
• Intimate partner violence and family conflict play a role at 24 months.
• Maternal depression and family conflict play a role at 36 months.

## 1 Introduction

Infants interact within a social world, and disruptions to the quality and quantity of these early interactions can affect their social cognition skills and socioemotional development (Choe et al., 2013, Pears and Moses, 2003, Rohrer et al., 2011, ﻿Ruffman et al., 1999; Wade et al., 2015; Winer and Thompson, 2011, ﻿Guajardo et al., 2009). Caregivers’ experience of a broad range of risk factors (e.g., negative life events, mental illness, high levels of family conflict, spousal violence, stress) reduces their own resources to provide supportive and sensitive caregiving (Choe et al., 2013, Lovejoy et al., 2000, Rohrer et al., 2011, Wade et al., 2015, Winer and Thompson, 2011, Campbell et al., 2004, Campbell et al., 2007), altering, in turn, a child’s social milieu. In support of the association between caregiver’s adverse experiences and children’s social skills evidence shows that maternal depression negatively affects social cognition, imitation and emotion recognition skills (Jensen et al., 2014, Perra et al., 2015, Rohrer et al., 2011, Winer and Thompson, 2011). Furthermore, maternal distress was shown to increase the risk of externalizing behaviours and decrease social skills (Choe et al., 2013, Jensen et al., 2014). Different measures of caregiving have been shown to mediate these associations including inductive discipline (i.e., the use of reasoning to explain parents' actions) and maternal warmth (Choe et al., 2013), maternal responsivity (Wade et al., 2015) and maternal negative and uninvolved interactions (Winer and Thompson, 2011). More specifically, rates of risk factors that are likely to alter a child’s early social environment, including mental illness, spousal violence, stress and family conflict, have been shown to be negatively correlated with socioeconomic status (SES), exposing the children living in low SES families to greater risk for atypical socioemotional developmental trajectories (Cunradi et al., 2000, Evans and English, 2002, Evans and Kim, 2010, Lund et al., 2010, Ridley et al., 2020, Sareen et al., 2011).

The link between SES and measures of social cognition and socioemotional development has been widely documented across the 20th century. Research following the Great Depression in the United States showed that economic loss was associated with increased stress and harsh caregiving, which predicted children’s socioemotional problems (e.g., Elder, 1979). More recently, low SES has been correlated with poor performance on social cognition tasks in preschoolers (Cutting and Dunn, 1999, Dunn et al., 1991, Pears and Moses, 2003) and an increased incidence of internalizing and externalizing behaviours in older children and adolescents (Evans and English, 2002, Duncan et al., 1994, McLoyd, 1990, McLoyd, 1997, McLoyd, 1998). In addition, a recent study that assessed cognitive and socioemotional development in 3–4 year old children in 35 low- and middle-income countries (LMICs) using the Early Childhood Development Index reported a higher prevalence of low scores in the socio emotional domain (26.2%) compared to the cognitive domain (14.6%; McCoy et al., 2016).

Recent efforts to use neuroimaging (i.e., fNIRS and EEG) to study the neural mechanisms through which low SES contexts impact brain development in infancy and early childhood have mostly focused on variables related to academic skills, such as executive functions and language development (Begus et al., 2016, Katus et al., 2020; Lloyd Fox et al., 2019; Tomalski et al., 2013; Wijeakumar et al., 2019). In contrast, fewer studies have investigated neural measures of social information processing (Lloyd Fox et al., 2017: Perdue et al., 2018). The focus on skills directly related to academic achievement is motivated by the real-world importance of the socioeconomic achievement gap, i.e. the disparity in academic achievement between students from high- and low- SES backgrounds (Farah, 2017). However, accumulating evidence shows that being in a lower SES may have an impact on one’s social emotional skills, therefore more work investigating neural markers of skills more closely related to social behaviour is warranted. Specifically, it is crucial for research to shed light on *when in development these effects on social cognition and socioemotional skills manifest* and *which risk factors contribute to these associations* in order to devise targeted interventions.

The social brain network represents an ideal candidate to interrogate, as its functions start to emerge during the first few months of life (Grossmann et al., 2015), and thus are vulnerable to the impact of early adversity. Exposure to altered social inputs (e.g., reduced parental engagement due to parental depression) or lower quality ones (either directly through suboptimal - harsh and unresponsive - caregiving, or indirectly by witnessing violence at home) could result in subtle functional changes in the brain that precede the onset of behavioural issues. Structural changes including reduced cortical thickness could also ensue from deprivation of social input (Mclaughlin et al , 2014). Therefore, measuring variability either in structural measures, functional responses or connectivity of the social brain network early in life and identifying those risk factors that are associated with this variability would be an initial step towards a mechanistic understanding of the link between low SES and compromised social development.

The current study employed functional Near Infrared Spectroscopy (fNIRS) to measure activation in regions of the brain associated with social processing in children growing up in a deeply impoverished neighbourhood in Dhaka Bangladesh. Data presented in this paper are part of a larger accelerated longitudinal design study, the Bangladesh Early Adversity Neuroimaging project (BEAN, https://www.lcn-bean.org/). The project’s overarching goal is to investigate the association between early risk factors and neurodevelopmental outcomes in two cohorts of children living in Dhaka, Bangladesh. Cohort 1 includes children that are assessed longitudinally at 2, 6, 24 and 60 months. Cohort 2 includes children assessed at 36 and 60 months. The study employs neuroimaging (MRI at 2 and 60 months; fNIRS and EEG at 6, 24, 36 and 60 months), eye tracking and physiological measures, alongside behavioural assessments and measures of quality of the child’s environment and caregiving practices. These data are complemented by a wealth of data on both biological (nutrition and inflammation measures) and psychosocial risk factors (e.g., questionnaire-based measures of adversity exposure and parental mental health). The current study presents data from one of the fNIRS tasks that was collected at 6, 24 and 36 months. Using near infrared light, fNIRS measures changes in concentration levels of oxygenated and deoxygenated haemoglobin. Neurovascular coupling warrants that this information on blood oxygenation is used to draw inferences on brain activity (Lloyd-Fox et al., 2010). Given its portability and adaptability to environmental conditions, over the past decade fNIRS has been increasingly and successfully implemented in studies conducted in LMICs (see Blasi et al., 2019 for a review), thus allowing researchers to complement existing research on neurodevelopment, which is by and large undertaken in high-income countries.

The goal of the present study is twofold: first we aimed to characterize neural responses over the inferior frontal to posterior temporal cortices in a well-established social discrimination task in two cohorts of children raised in a low-resource setting at three points in development: 6, 24 and 36 months of age. To this end, we used a social discrimination paradigm that has been successfully used with infants in both high- and low- income settings (Lloyd-Fox et al., 2007, 2009, 2017). Second, we aimed to relate these responses with psychosocial risk factors that we assessed at each timepoint using the Childhood Psychosocial Adversity Scale (CPAS) (Berens et al., 2019). The CPAS was devised to assess childhood psychosocial risk in a variety of cultural and socioeconomical settings, addressing the shortage of measures validated outside of high income, Western countries (Bick and Nelson, 2016). We focused on caregiver focused subscales of the questionnaire (social isolation, depression, intimate partner violence and verbal abuse and family conflict) as we hypothesized that by affecting caregivers’ resources for caregiving, these risks would directly reflect on children’s social environment, with their impact directly observable at the cortical level. Given that these risk factors can differentially shape a child’s early social experience, their individual contribution to social information processing was investigated. We measured how sensitive infants are in detecting the difference between social and non-social stimuli and we sought to test the hypothesis that increasing levels of parental exposure to psychosocial adversity would be associated with reductions in detecting such differences. Specifically, if the association between adversity and discrimination is by and large mediated by alterations in the direct interactions with the caregiver we expect it to emerge as early as 6 months given how extensively infants and parents interact during the first few months of postnatal life.

## 2 Materials and methods

### 2.1 Participants

Participants lived in an urban district (Mirpur neighbourhood) of Dhaka, Bangladesh characterized by profound poverty (monthly income: 30111 taka, 355 USD) and poor and unsanitary living conditions (e.g. unpaved streets, open sewages, poor housing materials (e.g. metal roofs), high levels of air and water pollution). Participants from two cohorts were enrolled before or at birth for BEAN. fNIRS data presented in this paper were collected during the visits at 6 and 24 months for Cohort 1, and at 36 months for Cohort 2. On the day of the visit all participants completed the fNIRS session and a cognitive assessment. At 24 months parent-child dyads also took part in a 5-minute free play session. fNIRS sessions included resting state and social discrimination paradigms for all time points; at 24 months the fNIRS session included an additional task.

At 6 months, 220 participants participated in the study (6.09 (*SD* 1.14) months old; female *n* = 120); 133 (60.5%) contributed data to the final analysis. Eighty-seven participants were excluded owing to: technical problems (N = 3); not completing the experiment (N = 46); an insufficient number of valid trials based on behavioural coding (N = 36); an insufficient number of valid trials following motion correction (N = 1); and a high number of rejected channels (N = 1).

At 24 months, 194 participants participated in the study (25.4 (*SD* 2.05) months old; female *n* = 110); 112 (57.7%) contributed data to the final analysis. Seventy-eight participants were excluded owing to: technical problems (N = 4); not completing the experiment (N = 26); an insufficient number of valid trials based on behavioural coding (N = 26); incorrect cap placement (N = 14); and a high number of rejected channels(N = 10).

At 36 months, 204 participants participated in the study (36.7 (*SD* 1.4) months old; female *n* = 89); 155 (76%) contributed data to the final analysis. Forty-nine participants were excluded owing to: technical problems (N = 6); not completing the experiment (N = 8); an insufficient number of valid trials based on behavioural coding (N = 12); and a high number of rejected channels (N = 23).

All infants were born full term (37–42 weeks’ gestation) and had no known neurological conditions, physical or neurological disability. Parents provided informed consent before enrollment in the fNIRS study.

### 2.2 Stimuli and design

**Fig. 1:**
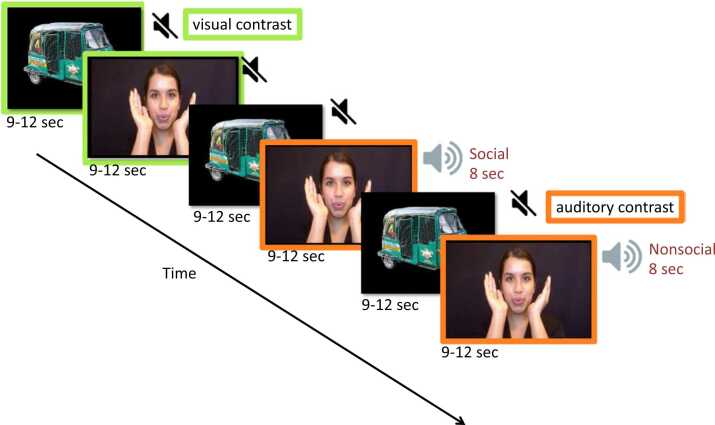
Experimental design. ﻿ The visual stimulus alternates between social and non-social stimuli (VN). The auditory stimulus is either silence (VS), social sounds (AS) or non‐social sounds (AN). Visual social discrimination was assessed measuring the response to the social silent stimulus (VS) relative to the non-social baseline (VN) (contrast highlighted in green). Auditory social discrimination was assessed comparing visual stimuli with social sounds (AS) and visual stimuli with non-social sounds (AN) (contrast highlighted in orange).

The experimental design and stimuli used for this study are the same as those used in Perdue et al. (2018) and have been described in detail elsewhere (Lloyd‐Fox et al., 2012, Lloyd‐Fox et al., 2014a). Briefly, visual social stimuli were short movie clips consisting of women performing nursery rhymes and peekaboo gestures. Visual nonsocial stimuli were pictures of methods of transportation, with seven pictures presented in each visual nonsocial (VN) trial. Both social and nonsocial stimuli were culturally adapted to be familiar to participants by showing women of South Asian descent, and modes of transportation typical in Bangladesh. One-third of visual social trials are presented with no sounds (visual social condition-VS), while during the remaining two-thirds either social (auditory social condition-AS) or non-social (auditory non-social condition-AN) sounds are played alongside the social video (see Fig. 1). Auditory stimuli were identical to those used in previous studies, with the social condition including communicative and non‐communicative non‐speech adult vocalizations (coughing, laughing, yawning, and crying), and the nonsocial condition including naturalistic environmental sounds likely to be familiar to infants and young children (e.g.: running water, rattles, etc.). Each experimental trial (VS, AN, AS) was followed by a baseline trial (VN). Trials were presented in the same order for every participant (VS-AN-AS-VS-AS-AN-VS-AS-AN-VS-AN-AS). At 6 and 36 months a maximum of 10 trials per experimental condition (VS, AN, AS) were presented, while at 24 months the maximum number of trials per condition was 6. The decision to reduce the total number of trials at 24 months lies in the longer experimental session for this age-group (three fNIRS tasks) compared to the other ones (two fNIRS tasks).

### 2.3 Apparatus

**Fig. 2:**
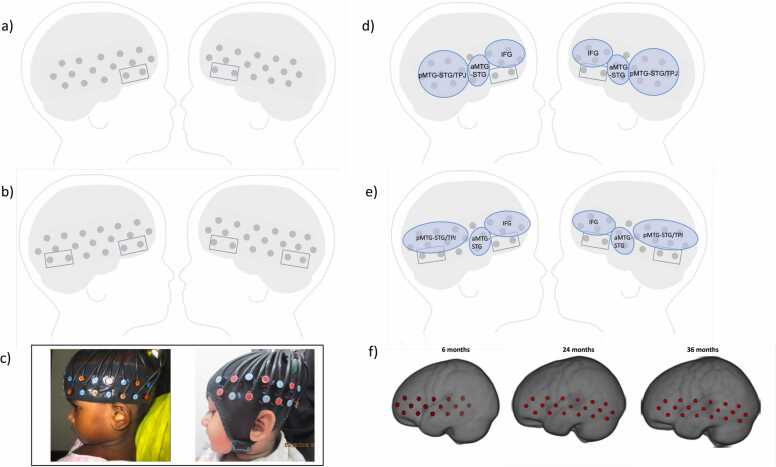
Schematic showing the channel location at 6 months (a) and at 24 and 36 months (b). Blue boxes indicate channels rejected due to cross talk issues. Panel c) shows the two headgear types used. At 6 and 36 months the headband (c,left) was used for every subject. At 24 months data was collected using both the headband and the EasyCap (c, right). Panels d) and e) show which channels contributing to each ROI at the group level at 6months (d) and at 24 and 36 months (e). Panel f) shows approximate position of channels on age specific templates.

fNIRS data were collected with the NTS optical topography system (Everdell et al., 2005). This system used 2 continuous wavelengths of source light at 780 and 850 nm. Participants wore custom-built headgear consisting of two source-detector arrays containing 32 channels at 6 months (12 sources and 12 detectors) and 38 channels at 24 and 36 months (14 sources and 14 detectors). This array is similar to the one used by Lloyd-Fox et al. in previous work with 10 sources and 10 detectors (e.g., Lloyd-Fox et al., 2017) and includes additional optodes at the back of each hemisphere. Source detector separation was 2 cm and the system sampling frequency was 10 Hz. The arrays were placed over both hemispheres and covered the inferior frontal - temporal lobes (see Fig. 2). At 24 months, for 28 participants an EasyCap was used instead of the custom build headband, and it was ensured that optode location was consistent across headgear types. After placing the cap on the child’s head pictures were taken from the front and the two sides and these were used to assess cap placement quality. If a shift larger than 1 cm relative to optimal placement was measured, a participant would not contribute data to the analyses.

### 2.4 Procedure

Before the study began, the experimenter measured the child’s head circumference and ear to ear lateral semi circumference. During the experiment, participants sat on their parent’s lap, approximately 1 m away from the screen where the stimuli were presented. Sounds were presented via two speakers embedded in the monitor of 3 W each. During sound presentation, sound amplitude ranged between 75 and 80 decibels. The parent was asked to refrain from interacting during the stimuli presentation unless the child became fussy or sought their attention. The experimenter stood behind the parent and the infant and helped to redirect the child’s attention towards the screen whenever necessary. If the child grew too distracted or fussy, stimulus presentation was paused and resumed after a short break. The experiment ended either when the max number of experimental trials was reached or when the child became fussy. Each session was recorded using a video camera placed just above the screen, and infant behaviour and experimenter interference were coded offline.

### 2.5 fNIRS data preprocessing

We first assessed the quality of the data using the toolbox QT_NIRS (https://github.com/lpollonini/qt-nirs, Hernandez and Pollonini, 2020). This step revealed that 4 channels over the bottom-front in the 6mo group and 8 channels over the bottom-front and bottom-back in the older age groups were affected by an intermittent pattern of correlation/anticorrelation of the two wavelengths (see Fig. 2b for the position of these channels). Cross talk of the encoding frequency of the sources of light paired with the same detector in the NIRS array was identified as the main cause of this anomalous pattern, which remarkably altered the neurophysiological signal, without any possibility of recovering it. Therefore, these channels were removed from further analyses for every participant.

Following this step, data were preprocessed using the Homer2 (Huppert et al., 2009) toolbox for MATLAB (The Mathworks, Natick, MA). First channels with very low optical intensity readings (mean below 0.003 V) were excluded and participants with less than 2/3 valid channels were not included in further analyses. Raw intensity data was converted to optical density. Motion artifacts in the data were corrected using a combination of spline interpolation (Scholkmann et al., 2010) and wavelet filtering (Molavi and Dumont, 2012), following recent work that suggests that the combination of these approaches outperforms their individual use for the analysis of developmental datasets (Di Lorenzo, Pirazzoli et al., 2019). Motion artifacts were first identified as those portions of signal exceeding a threshold in change of amplitude (0.4) and a threshold in change of standard deviation (15) within a predefined time-window (1 s) and data points around the detected motion (1 s) marked. Spline interpolation was then applied channel by channel to these preidentified artifacts, followed by the wavelet motion correction algorithm (iqr=0.8). Motion detection was applied to identify the remaining uncorrected motion artifacts and trials affected by it (−2 to 16 s relative to trial onset) were rejected.

Experimental trials were also rejected based on behavioural coding performed offline using video recordings. Two criteria were used: a trial was rejected if a subject did not attend at least 60% of it and if social interference (interaction between experimenter/parent and child) occurred during the experimental trial or the previous baseline. A subject was included in the analyses only if they contributed at least 3 valid experimental trials per condition. The average number of valid trails following application of the different exclusion criteria was: at 6 months, 6.2 (62%) visual social trials, 6.3 (63%) auditory social trials, 6.3 (63%) the auditory non-social trials; at 24months, 4.2 (70%) visual social trials, 4.8 (80%) auditory social trials, 4.8 (80%) auditory non-social trials; at 36months, 8.1 (81%) visual social trials, 8 (80%) auditory social trials, 7.5 (75%) auditory non-social trials.

A bandpass filter was then applied from 0.03 Hz to 1 Hz to reduce slow drifts and high-frequency noise. Data was converted from optical density to oxygenated (HbO_2_) and deoxygenated (HHb) haemoglobin concentrations using the modified Beer Lambert Law (partial path factor (ppf)= 5.1 at 6 months; ppf= 6 at 24 and 36 months). During this step data for each participant and each channel was segmented into blocks of 22 s of data, including 2 s pre-stimulus onset, a 10 s experimental trial, plus the following 10 s baseline trial, using the pre-stimulus time for baseline correction. Valid trials for each experimental condition *(VS, AS, AN)* were averaged together for each infant, and a time course of the mean change in HbO_2_ and HHb concentration changes was compiled for each channel (for group level channel-by-channel time courses see Supplementary Material, Fig. S1, S2, S3).

### 2.6 Anatomical localization

Anatomical localization of the optodes’ locations was performed at the subject level, to account for differences in head size and array placement. Participants were matched to individual MRIs in the Neurodevelopmental database (Richards et al., 2016; Sanchez et al., 2012) by age and head measurements (head circumference, ear‐to‐ear measurements over forehead and over the top of the head). A previously developed algorithm was used to place a virtual array on the MRI head model (Fu and Richards, 2021). For each participant, the placement of the array was checked and, in case of inaccuracies, adjusted to match the photographs of the participant during the experiment. If accurate optode locations could not be achieved on the matched MRI, a new MRI was selected based on matching just the ear‐to‐ear measurements over the forehead of the child and the MRI. Photon propagation modeling (Fang, 2010) was used to estimate the diffuse optical tomography sensitivity functions from each source and detector pair comprising a channel. The intersecting cortex regions were labeled using an atlas created for this study. Since this task has been previously shown to elicit selective activations over ﻿regions including inferior frontal gyrus (IFG), anterior middle temporal gyrus/superior temporal gyrus (aMTG-STG), and posterior superior temporal sulcus/temporoparietal junction (pSTS-TPJ), we generated an atlas with these three ROIs, combining data from different existing atlases. pSTS-TPJ was calculated by grouping together the posterior portion of STG and MTG and the TPJ and will henceforth be referred to as pMTG-STG/TPJ. The ROIs were defined using the participant-based-atlases (LPBA, Hammers, Brainnetome), choosing the appropriate atlas-ROI area, and using any atlas ROI that defined the ROI. For example, IFG was generated combining LPBA40, (25 L_inferior_frontal_gyrus; 26 R_inferior_frontal_gyrus) Hammers, (56 Inferior_frontal_gyrus_left, 57 Inferior_frontal_gyrus_right) and Brainnetome, (29 IFG6_1_A44d_L, 30 IFG6_1_A44d_R, 31 IFG6_2_IFS_L, 32 IFG6_2_IFS_R, 33 IFG6_3_A45c_L, 34 IFG6_3_A45c_R, 35 IFG6_4_A45r_L, 36 IFG6_4_A45r_R, 37 IFG6_5_A44op_L, 38 IFG6_5_A44op_R, 39 IFG6_6_A44v_L, 40 IFG6_6_A44v_R) data. The TPJ was defined as the intersection of the STG, angular, and supramarginal gyri. Each of these ROIs was first defined from the atlas-ROI, dilated by 3 mm, and the conjunction of the areas was defined as the TPJ (Lloyd Fox et al., 204b). For each participant using diffuse optical tomography data, we estimated the sensitivity of each channel to each ROI. In order to group channels into ROIs a channel was assigned to an ROI if it’s sensitivity to that ROI exceeded 50%. This threshold was selected so that each single channel would be largely sensitive to one ROI only. ROIs were calculated at the individual level to account for differences in cap placement and head circumference, thus different channels could contribute to the same ROI across participants.

Three brain ROIs in each hemisphere could be measured in most participants—left and right inferior frontal gyrus (lIFG, rIFG), left and right posterior middle temporal gyrus-superior temporal gyrus/TPJ (lpMTG-STG/TPJ, rpMTG-STG/TPJ), and left and right anterior middle temporal gyrus/superior temporal gyrus (laMTG-STG, raMTG-STG). See Fig. 2 for the approximate location of the channels for each ROI at 6 (Fig. 2d), 24 and 36 months (Fig. 2e). Details on number of channels included in in each ROI can be found in the Supplementary Material in Table 1 and in Fig. S4.

**Table 1:** Social discrimination in each ROI for the visual and auditory domains. p values marked in bold are < 0.05. Asterisks are next to p-values that survived FDR corrections.

	visual					
	*HbO2*					
	6mo		24mo		36mo	
	t(df)	p	t(df)	p	t(df)	p
rIFG	3.697 (117)	< 0.001 *	4.786 (107)	< 0.001 *	5.617 (152)	< 0.001 *
raMTG/STG	1.366 (81)	0.176	2.081 (34)	**0.045**	0.993 (91)	0.323
rpMTG-STG/TPJ	6.103 (132)	< 0.001 *	8.998 (106)	< 0.001 *	8.332 (138)	< 0.001 *
lIFG	3.735 (124)	< 0.001 *	4.05 (108)	< 0.001 *	4.032 (144)	< 0.001 *
laMTG/STG	2.005 (74)	**0.049**	0.013 (44)	0.99	-0.08 (116)	0.936
lpMTG-STG/TPJ	4.772 (118)	< 0.001 *	8.446 (108)	< 0.001 *	4.551 (137)	< 0.001 *
	*HHb*					
rIFG	-0.378 (117)	0.706	0.464 (107)	0.643	-0.924 (152)	0.356
raMTG/STG	-0.562 (81)	0.575	-0.975 (34)	0.336	-0.907 (91)	0.366
rpMTG-STG/TPJ	-1.277 (132)	0.204	-0.206 (106)	0.836	-1.507 (138)	0.133
lIFG	0.512 (124)	0.609	-0.421 (108)	0.674	-1.504 (146)	0.134
laMTG/STG	1.044 (74)	0.300	0.0841 (44)	0.933	0.204 (116)	0.838
lpMTG-STG/TPJ	1.024 (118)	0.308	-0.738 (108)	0.461	-1.816 (137)	0.071
	**auditory**					
	*HbO2*					
	6mo		24mo		36mo	
	t(df)	p	t(df)	p	t(df)	p
rIFG	0.339 (117)	0.735	3.34 (107)	0.001 *	0.932 (152)	0.353
raMTG/STG	1.15 (81)	0.254	1.604 (34)	0.118	0.013 (91)	0.99
rpMTG-STG/TPJ	1.975 (132)	0.05	1.873 (106)	0.064	0.625 (138)	0.533
lIFG	2.361 (124)	**0.02**	1.479 (108)	0.142	1.692 (144)	0.093
laMTG/STG	1.45 (74)	0.151	0.083 (44)	0.934	1.661 (116)	0.099
lpMTG-STG/TPJ	2.034 (117)	**0.044**	1.155 (108)	0.251	0.276 (137)	0.783
	*HHb*					
rIFG	0.190 (117)	0.849	-0.094 (107)	0.924	-0.963 (152)	0.336
raMTG/STG	-1.71 (81)	0.089	0.616 (34)	0.541	1.185 (91)	0.238
rpMTG-STG/TPJ	-0.996 (132)	0.321	-0.262 (106)	0.793	-0.872 (138)	0.384
lIFG	-1.882 (124)	0.062	-3.144 (108)	0.002	-0.743 (144)	0.458
laMTG/STG	0.448 (74)	0.655	0.931 (44)	0.356	-1.939 (116)	0.054
lpMTG-STG/TPJ	-0.158 (117)	0.873	-0.368 (108)	0.712	-1.108 (137)	0.269

### 2.7 Childhood psychosocial adversity scales (CPAS)

The Childhood psychosocial adversity scales (Berens et al., 2019) were administered at the time of the neurocognitive assessment for each cohort. At 6 months and 36 months, CPAS data is available only for a subgroup of participants (6 months, n = 109; 36 months, n = 76). The reason for the smaller sample size at these timepoints is that the CPAS was tested and validated at the onset of the BEAN project, and it was ready to be implemented in the study halfway through data collection within these cohorts. At the 24 months timepoint, CPAS was collected from the entire cohort (n = 196). The CPAS includes nine subscales divided in child-, caregiver- and household/community- focused subscales. For this work we used scores from the four caregiver-focused subscales: depression, social isolation, physical intimate partner violence, verbal abuse and family conflict.

### 2.8 Analysis plan

Data were averaged in six ROIs (right and left IFG, right and left anterior MTG-STG, right and left posterior MTG-STG/TPJ) following anatomical localization. Within each ROI the brain response was averaged between 12- and 16-seconds post stimulus onset to include the range of maximum concentration changes for HbO_2_ and HHb. This time window was selected in line with prior studies using this stimulus (Lloyd‐Fox et al., 2017, 2018) and was confirmed following visual inspection of the time-courses across all channels (Fig. S5).

To identify those ROIs that showed a visual social discrimination, statistical comparisons (paired t-tests) were performed, to compare the averaged signal over the selected time window during the visual social condition, with the averaged pre-stimulus signal (i.e., last two seconds of baseline). Either a significant increase in HbO_2_ and/or a significant decrease in HHb is interpreted as cortical activation. To identify ROIs where auditory social discrimination occurred, paired t-tests were used for the direct comparison of the auditory social and non-social conditions during the specified time windows. To account for errors due to multiple comparisons, p-values were corrected using a MATLAB false discovery rate (FDR) function (Benjamini and Hochberg, 1995). To test the effects of ROI, hemisphere, and age on visual and auditory social discrimination we employed a relative measure (percentage of participants with significant response). The use of a relative measure was necessary because in the model estimating effects on visual social discrimination the dependent variable is the amplitude of hemoglobin in response to the visual social condition; amplitude measures cannot be directly compared across different ROIs, due to the DPF being region specific (Zhao et al., 2002), and across different ages, due to the DPF being only a rough estimate value. For consistency purposes we applied the same analysis also to the social auditory discrimination measure even if auditory discrimination is calculated as the relative difference in amplitude between two conditions (social and non-social auditory), which should be independent of the DPF. This analysis can be found in the Supplementary Materials.

To facilitate comparisons with previous studies using the same task we also performed channel-by-channel analyses. The same analytical approach described for the ROIs was applied to individual channels. Results are presented in the Supplementary Materials (Figs. S6, S7, S8).

Finally, multiple linear regression was performed to test the associations between the CPAS subscales and the neural measures of social information processing. In the regression model, the different CPAS subscales were entered together as predictors and the dependent variable was the visual (HbO_2_ amplitude averaged 12–16 s following onset of the visual social condition) or auditory social contrast (difference in averaged HbO_2_ amplitude between social and non-social auditory conditions) at each ROI in each age group. At 24 months all four subscales were used as predictors, while at 6 and 36 months physical intimate partner violence was dropped from the model due to a lack of variability in this measure (see Table 2 for details). Besides testing associations within age group, we also tested longitudinal association within Cohort 1. Specifically, using multiple linear regression we tested whether psychosocial adversity measured at 6 months predicted neural responses at 24 months, and using simple correlations we explored whether neural responses at 6 months are associated with CPAS at 24 months.

**Table 2:** CPAS caregiver focused subscales’ statistics in each age group.

subscale	age (months)	Mean	SD	Min	Max	Cronbach's α
**social isolation***(items: 5; max score: 20)*	6	1.825	3.27112	0	14	0.864
	24	3.4975	5.04634	0	20	0.949
	36	2	3.62806	0	17	0.864
**intimate partner violence***(items: 7; max score: 28)*	6	0.3083	1.05158	0	7	0.746
	24	1.3858	3.32329	0	20	0.909
	36	0.3333	1.15805	0	8	0.782
**verbal abuse and family conflict***(items: 5; max score: 20)*	6	2.4417	2.45222	0	13	0.687
	24	4.9848	3.99167	0	17	0.861
	36	2.7241	2.54124	0	12	0.635
**maternal depression***(items: 8; max score: 32)*	6	4.6417	5.16093	0	26	0.882
	24	6.3909	6.9996	0	28	0.945
	36	5.5862	6.14657	0	24	0.89

## 3 Results

### 3.1 Neural responses

#### 3.1.1 Social discrimination

For each cohort visual and auditory social discrimination (i.e., the ability to detect the difference between social and non-social stimuli) was measured for each ROI and chromophore (see Table 1 for complete statistics and FDR corrected *p*-values).

At **6 months** social discrimination in the visual domain (i.e., a significant increase in HbO_2_ during the visual social condition relative to baseline) was observed in right IFG (*t*(117) = 3.96,*p* < .001), right pMTG-STG/TPJ (*t*(132) = 6.1, *p* < .001), left IFG(*t*(124) = 3.73,*p* < .001), left aMTG/STG (*t*(74) = 2, *p* = .049) and left pMTG-STG/TPJ (*t*(118) = 4.7, *p* < .001) in HbO_2_. Social discrimination in the auditory domain (i.e., greater HbO_2_ response to the auditory social compared to the auditory non-social condition) was observed in left IFG (*t*(124) = 2.36 *p* = .02), left pMTG-STG/TPJ (*t*(117) = 2.03, *p* = .044) and marginally in right pMTG-STG/TPJ (*t*(132) = 1.9, *p* = .05) in HbO_2_.

At **24 months** social discrimination in the visual domain was observed in right IFG (*t*(107) = 4.78,*p* < .001), right pMTG-STG/TPJ (*t*(106) = 9, *p* < .001), left IFG(*t*(108) = 4.05,*p* < .001) and left pMTG-STG/TPJ (*t*(108) = 8.44, *p* < .001) in HbO^2^. Social discrimination in the auditory domain was observed in right IFG (*t*(107) = 3.34, *p* = .001) in HbO^2^ and in left IFG (*t*(108 = −3.44, *p* = .002) in HHb.

At **36 months** social discrimination in the visual domain was observed in right IFG (*t*(152) = 5.61,*p* < .001), right pMTG-STG/TPJ (*t*(138) = 8.33, *p* < .001), left IFG (*t*(144) = 4.03,*p* < .001) and left pMTG-STG/TPJ (*t*(137) = 4.55, *p* < .001) in HbO^2^. Social discrimination in the auditory domain was not observed in any ROI.

In Supplementary Materials (Fig. S6-S8) ROI results are presented alongside the channel-by-channel analyses. Though channel by channel analyses are not used for the main analyses, these are reported for a more direct comparison with previous work using this task.

### 3.2 Association with adversity

Descriptive statistics for the caregivers’ focused subscales can be found in Table 2. For families from Cohort 1 that completed the CPAS both at the 6 and at the 24 months timepoint, scores increased significantly overtime for the intimate partner violence (*t*(90) = −2.34, *p* = .021) and verbal abuse and family conflict subscales (*t*(90) = −4.13, *p* < .001). At recruitment scores for the two cohorts (6 and 36 months) did not significantly differ for any of the subscales (all *p*s. >.23).

**﻿**Multiple linear regression analyses were performed to examine the associations of neural measures of social discrimination with psychosocial adversity in each age-group, separately for each ROI.

At **6 months**, the analyses showed no effect of the different caregiver focused subscales of the CPAS on neural measures of social discrimination in any of the ROIs.

**Fig. 3:**
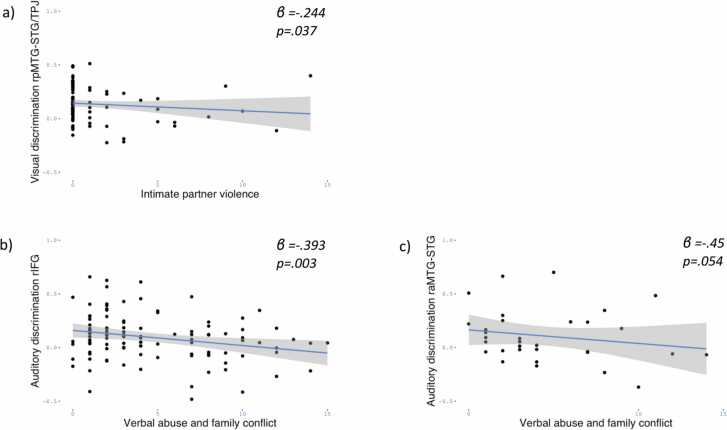
Regression lines 24 months. Panel a: linear association between intimate partner violence and visual discrimination in left pMTG-STG/TPJ. Panel b: linear association between verbal abuse and family conflict and auditory discrimination in right IFG. Panel c: linear association between verbal abuse and family conflict and auditory discrimination in right aMTG/STG. The shade areas represent the 95% CI of the regression line.

At **24 months**, intimate partner violence and verbal abuse and family conflict emerged as predictors of the magnitude of social discrimination**.** The overall regression model for the visual social discrimination in left pMTG-STG/TPJ approached significance *F*(4, 102) = 1.8, *p* = .133, *R*^*2*^ = .066, while intimate partner violence was a significant predictor of visual social discrimination in left pMTG-STG/TPJ (*β* = −.244, *t* = −2.11, *p* = .037) (Fig. 3a).

The overall regression model for the auditory social discrimination in right IFG was significant *F*(4, 101) = 2.48, *p* = .049, *R*^*2*^ = .089, and verbal abuse and family conflict was a significant predictor (*β* = −.393, *t* = −3.05, *p* = .003) (Fig. 3b). Verbal abuse and family conflict was also a marginally significant predictor for auditory social discrimination in right aMTGSTG (*β* = −.45, *t* = −2.005, *p* = .054) despite the lack of significance of the overall model, *F*(4, 30) = 1.12, *p* = .364, *R*^*2*^ = .13 (Fig. 3c).

No associations with CPAS subscales were found for other ROIs.

Overall, at 24 months we observed negative associations between adversity and social discrimination. Fig. 5 (top panel) depicts a summary of these findings.

**Fig. 4:**
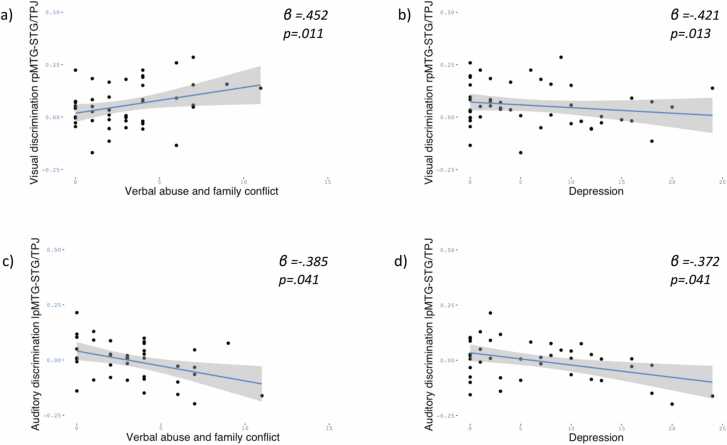
Regression lines 36 months. Panel a: linear association between verbal abuse and family conflict and visual discrimination in right pMTG-STG/TPJ. Panel b: linear association between depression and auditory discrimination in right pMTG-STG/TPJ. Panel c: linear association between verbal abuse and family conflict and auditory discrimination in right pMTG-STG/TPJ. Panel d: linear association between depression and auditory discrimination in left pMTG-STG/TPJ. The shade areas represent the 95% CI of the regression line.

At **36 months**, depression and verbal abuse and family conflict emerged as predictors of the magnitude of social discrimination. The overall regression model for the visual social discrimination in right pMTG-STG/TPJ was significant *F*(3,46)= 4.35, *p* = .009, *R*^*2*^ = .22, and verbal abuse and family conflict (*β* = .452, *t* = 2.66, *p* = .011) and depression (*β* = −.421, *t* = −2.57, *p* = .013) were 0.013 predictors. (Fig. 4a,b).

The overall regression model for the auditory social discrimination in left pMTG-STG/TPJ was significant *F*(3,37)= 4.27, *p* = .01, *R*^*2*^ = .25, and verbal abuse and family conflict (*β* = −.385, *t*[TS−0.385= −2.119, *p* = .041) and depression (*β* = −.372, *t* = −2.117, *p* = .041) were significant predictors (Fig. 4c,d).

No associations with CPAS subscales were found for other ROIs.

**Fig. 5:**
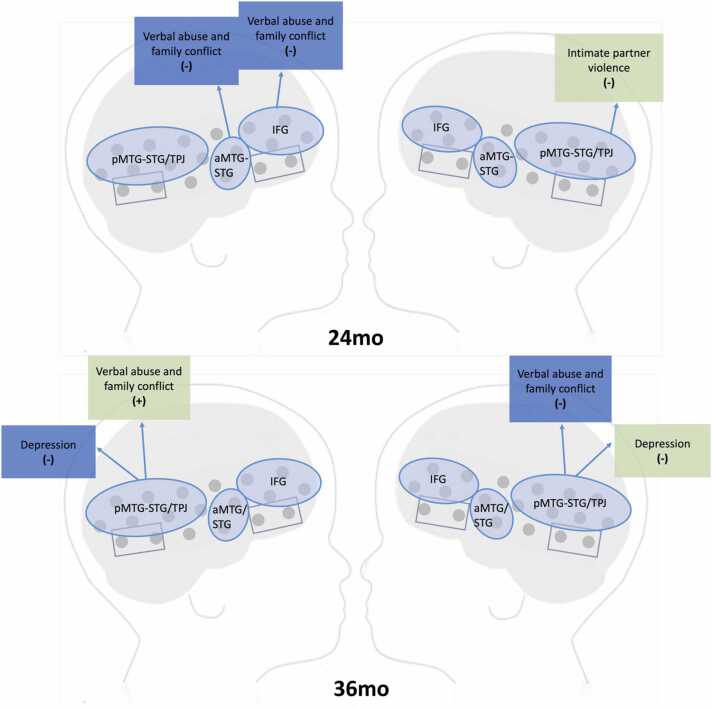
schematic depicting summary of associations between CPAS subscales and auditory (blue boxes) or visual (green boxes) social contrast magnitude in 24 and 36 months children sign in brackets indicates direction of association.

Overall, at 36 months we observed negative associations between adversity and social discrimination, except for one positive association between verbal abuse and family conflict and visual discrimination in left pMTG-STG/TPJ. Fig. 5 (bottom panel) depicts a summary of these findings.

Testing associations between **6 and 24 months** measures, we found that none of the CPAS subscales measured at 6 months predicted social discrimination at 24 months, and that there were no significant correlations between social discrimination at 6 months and CPAS at 24 months.

## 4 Discussion

The aim of the present work was to investigate the development of cortical social selectivity in children raised in a low SES setting and to identify psychosocial risk factors that could alter this basic form of social information processing. The most important finding from this work is that caregivers’ experience of psychosocial adversity, including depression, intimate partner violence and family conflict, is shown to impact toddlers’ neural discrimination of social stimuli.

Using fNIRS we measured responses to visual and auditory social and non-social stimuli at 6, 24 and 36 months over cortical regions known to be selective to social stimuli from infancy. For visual stimuli we report a consistent pattern of findings, with IFG and pMTG-STG/TPJ bilaterally displaying social selectivity across all age groups.

This finding is consistent with previous work using the same task. Indeed, whilst most previous work used a channel-by-channel analysis approach, social selectivity was reported over channels that were identified to cover IFG and pMTG-STG/TPJ regions (Lloyd-Fox et al., 2017, Lloyd‐Fox et al., 2009, Lloyd‐Fox et al., 2019, Lloyd‐Fox et al., 2013). In order to facilitate comparisons across studies, channel by channel analyses were performed as well as part of the present work. From these, it emerged that at 6 months the extent of visual social selectivity (Fig. S6-S8) is broadly comparable to that observed at similar ages across UK and Gambian infants by Lloyd-Fox and colleagues (i.e., broad responses to visual social information across IFG and temporal areas; Lloyd-Fox et al., 2009, 2013, 2017). However, at 24 months differences emerge when compared with data collected in the 18–24 months old cohort from The Gambia (though it is important to note that data collection was limited to one hemisphere in this study). While significant visual social selectivity in the Gambian cohort was confined to one channel over right pMTG-STG/TPJ, it was seen in most bilateral channels in our cohort. One possible explanation for this discrepancy is that the larger sample employed here (N = 112 vs. N = 19) increased power to detect smaller effects that may emerge at 2 years (in line with this, note that more widespread responses have now also been observed in a new cohort of N = 128 in The Gambia at 24 months of age, Bulgarelli et al., in prep). Interestingly, channel by channel analyses suggest that the extent of social selectivity is more widespread at 24 months relative to at 6 and 36 months; showing significant responses in 34 out of the 38 channels and thus evident both within and outside of the channels that contribute to the ROIs. Though this question was beyond the scope of the current work, future analyses could investigate whether specific elements of the environment that children in our two different cohorts were exposed to have influenced specialization to social visual stimuli.

As far as auditory social selectivity is concerned, we show a less consistent pattern of responses. Social selectivity within ROIs to auditory stimuli was measured at 6 months over the left pMTG-STG/TPJ and IFG (though it did not survive FDR corrections), at 24 months over the right IFG, and could no longer be detected at 36 months. Channel by channel analyses are also less consistent (relative to the visual social responses) with the ROI analyses. Firstly, they instead suggest that specialization to auditory social stimuli is lateralized to right channels (when FDR corrected) or left channels when not FDR corrected (in line with the ROI results). Secondly, they reveal a shift between 6 and 24 months when a more widespread and robust bilateral social selectivity (a higher number of channels, which also survive FDR correction) is observed over anterior temporal and frontal regions (right only) in the older age group. Furthermore, there is some suggestion of auditory social selectivity at 36 months, though these responses do not survive correction for multiple comparisons. These findings stand in contrast with previous work in two ways. First, the extent of auditory selectivity at the channel level at 24 months is much broader than the one reported in 18–24 month old children in The Gambia (Lloyd-Fox et al., 2017). Second, in our analyses the anterior temporal ROI did not show auditory social selectivity whilst social auditory selectivity has been localized to this cortical region by Lloyd-Fox and colleagues following anatomical co-registration in UK 4 – 7 month olds (Lloyd-Fox et al., 2014b). One possible reason for this discrepancy is that the anterior MTG/STG ROI could only be identified during anatomical registration in 34 (right hemisphere) and 44 (left hemisphere) participants out of 112 at 24 months (i.e., we could identify channels with sensitivity to this ROI greater than 50% in less participants compared with the other ROIs). The smaller sample size for this ROI may have hindered the detection of the effect. It is possible that as a result of development, scalp to cortex locations changed and that this ROI lies at the periphery of the array in this age group. Furthermore, given that the anterior temporal ROI lies adjacent to the Sylvain fissure it is possible that this ROI is more challenging to identify within individuals using this method – and that the channel by channel analysis (which does not confine itself to single ROIs) may have more accurately revealed activation patterns in this region. Alternatively, it is possible that the anterior temporal ROI was not accurately captured given that the co-registration was performed with MRIs from US children. Whilst we think this was the best available approach to identify cortical regions and group channels into ROIs, either MRIs from the same participants (as performed in Lloyd-Fox et al., 2014b) or from matched Bangladeshi children would have represented the optimal choice for co-registration. Indeed, previous research has shown morphometry differences, for both brain and head, between different ethnic groups and suggested the use of appropriate atlases (i.e., Caucasian and Asian; Tang et al., 2010, Tang et al., 2018; Xie et al., 2015). Future work should quantify differences using MRIs from the same ethnic group of our participants to clarify whether this finding is the effect of development, the effect of trying to localize a small ROI near to the Sylvain fissure or of suboptimal co-registration processes.

Differences in auditory social selectivity patterns between this and previous works could not be explained with differences in behavioral engagement with the stimuli, as the percentage of trials included per condition following behavioral coding was similar across studies. Instead, we believe that the auditory environment (both during the testing sessions and more broadly during daily life) should be considered as a potential explaining factor. The lab in which these data were collected does not have the advantage of being soundproof, and noises from the street could therefore be heard in the testing room (for an in-depth discussion on the comparison between well-resourced western labs and LMIC based labs, see Blasi et al., 2019). Given the variable nature of this interference (noise can come from vehicles, crowds visiting the local market a nearby factory and nearly constant construction sites) and the ensuing difficulty to measure it, we did not control for it and therefore cannot quantify its impact on our findings. While contamination of noise during the task is the most likely explanation for these discrepancies, we can’t rule out that children’s daily exposure to noise pollution could have also had an effect. In Mirpur, the neighborhood where families for the study have been recruited from, noise levels have been reported to be generally high and often exceed permissible levels (e.g., Chowdhury et al., 2010), and indoor spaces are not optimally screened from outdoor noise (due for example to poor construction materials, high temperatures that require opening windows, metal roofs). Of relevance to the current work, daily exposure to high noise levels could have altered the ratio of the non-social vs. social auditory inputs experienced by children in our study (relative to children raised in more affluent western contexts or in rural contexts, such as The Gambia) and as a consequence the developmental trajectory of cortical specialization to auditory social stimuli.

Central to our study was the question on how exposure to psychosocial adversity impacts social information processing. Overall, our findings support the hypothesis that psychosocial adversity negatively impacts this basic form of social cognition. Consistent with previous studies on the same cohorts, we did not find associations with measures of adversity at 6 months, lending further support to the idea that effects of adversity emerge with age, (Xie et al., 2019a, Xie et al., 2019b, Jensen et al., 2021). At 24 and 36 months higher levels of verbal abuse and family conflict predict poorer social auditory discrimination. This measure of family conflict could exert its effects on social information processing either through changes in caregiving such as a decrease in sensitivity (Krishnakumar and Buehler, 2000), or through exposure to an altered auditory environment such as aggressive interactions between caregivers. Work on young adults showed that exposure to verbal abuse during childhood is associated with altered gray matter volume in left the superior temporal gyrus and reduced structural connectivity in the left arcuate fasciculus (Tomoda et al., 2011, Choi et al., 2009).

This raises the possibility that the impact of exposure to (direct or indirect) verbal abuse on the functional measures observed in this study is underpinned by the early stages of structural alterations reported in young adults.

Extensive work suggests that parental conflict is associated with socioemotional problems during childhood (Cummings and Davies, 2010, Davies et al., 2007). To date, the impact of exposure to interparental conflict early in development on neural markers of social cognition has been investigated in one study using fMRI. The authors found that higher levels of conflict are associated with increased neural responses to angry vs. neutral speech in 6- to 12-month-old children in the anterior cingulate cortex, thalamus, caudate and hypothalamus (Graham et al., 2013). Our data converge with previous research in suggesting that family conflict plays a critical role in shaping early brain responses to social stimuli. While we detected this effect starting at 24 months, we cannot exclude that it could be detected earlier (i.e., between the ages of 6 and 24 months), in brain regions that were not measured due to limitations of the current optode layout (e.g. prefrontal cortex) or due to limitations of fNIRS measurements (i.e. subcortical structures cannot be measured once infants reach a few weeks of age).

Another robust and important result of this work is that depression emerges as a predictor of decreased social auditory discrimination at 36 months. The latency of this effect suggests that it may not be solely mediated by reduced caregiving quality, but that maternal depression could affect other aspects of the child’s psychosocial environment and the ensuing cumulative effects either become prominent around 36 months or increase overtime until they can be detected at 36 months. However, we need to be cautious in over interpreting that these are age specific, rather than cohort specific, differences given that the 36-month data represents a different cohort to the one that provided data at 6 and 24 months.

These data also suggest that visual social discrimination is impacted to a lesser extent compared to auditory social discrimination: increased visual social discrimination in pMTG-STG/TPJ is associated with lower scores on the intimate partner violence subscales at 24 months and with higher scores on the verbal abuse and family conflict subscale at 36 months. An unexpected and challenging to interpret finding of this work is that exposure to verbal abuse and family conflict seems to affect discrimination in the two sensory domains in opposite ways at 36 months (negative association with auditory, positive with visual discrimination). Whilst not in line with the initial hypotheses, this finding could be situated within the broader and fast-growing literature that explores the enhanced skills of children raised in high adversity contexts (Ellis et al., 2020). One hypothesis advanced in this research field is that people living in low SES contexts are more attuned to other people and social information. A possible explanation is that the due to unpredictability of their environments they learn to attend preferentially to people who can influence their life outcomes (Kraus et al., 2012). While evidence in support of this hypothesis comes from work with adults (see Ellis et al., 2020 for a review) it is possible that for the same reason unpredictable caregiving could lead children, whose survival depends on their caregivers, to become more attuned to social stimuli. Future work in this area will elucidate the differential impact (both positive and negative) that exposure to high adversity contexts early in development has on social skills.

These results show important similarities with recent work on a subset of the fNIRS data used in this study (Perdue et al., 2019) despite large differences across studies (i.e., sample size, age groups included, preprocessing of the fNIRS data, ROIs selection and calculation, risk factors explored in association to social information processing). Indeed, in Perdue et al., the effect of psychosocial adversity (a composite score including caregiver’s perceived stress and depression scores) could also be detected at 36 but not at 6 months. Despite measures of depression differing across the two studies (﻿Edinburgh Postnatal Depression Scale vs. CPAS) these results converge to show that depression takes longer to manifest its effects on social information processing. Furthermore, both works show that psychosocial adversity has a more evident impact on the auditory domain relative to the visual domain, which seems to be largely spared. Overall, the latency of the effects observed in this study could suggest that while caregiver’s experiences may already impact typical developmental trajectories in infancy, their effects are not obvious until 24 months. Although, in this work we did not find any evidence of long term effects of adversity on social discrimination (by testing whether CPAS measures at 6 months predict social discrimination at 24 months), the smaller sample size and less variability in CPAS scores at 6 vs. 24 months could have hindered this effect. Future work should strive to elucidate the role that adversity in early developmental stages has on later development in order to inform timely and effective interventions, and to include measures of caregiving practices and more broadly of the child’s social environment, which will promote understanding of the mechanisms behind these associations. Given the modest effects of the regression models (R^2^s < .22), future work should also strive to pinpoint other factors or combination of factors that explain a larger portion of the variance.

Importantly, by detecting the detrimental effects of psychosocial adversity on a basic measure of social discrimination, findings from this work show the need to complement existing research that uses neuroimaging to quantify the impact of low resource settings on brain development with measures pertaining socio emotional development. By employing tasks tapping into different aspects of social cognition (e.g., emotion processing and theory of mind) together with outcome measures related to socioemotional development, future research should aim to 1) elucidate the mechanisms through which socio emotional development is compromised and 2) gain a more comprehensive understanding of the impact that exposure to early life adversity has on both cognitive and socioemotional skills.

## **Conclusion**

In conclusion, these data show that while in infancy social information processing is by and large spared by caregiver’s adverse experiences, social discrimination in toddlers is associated with measures of intimate partner violence, verbal abuse and parental conflict, and depression. This is the first study to successfully implement and show predictive power of the CPAS, highlighting the importance of using measures of adversity developed and tailored to the cultural context where data is to be collected. Leveraging the subscales scores, compared to the total score, afforded us a more fine-grained understanding of the differential impact of different forms of psychosocial adversity, opening opportunities for targeted interventions.

## Funding source

10.13039/100000865Bill and Melinda Gates Foundation (OPP1111625).

## Financial disclosure

The authors have no financial relationships relevant to this article to disclose.

## Declaration of Competing Interest

The authors declare that they have no known competing financial interests or personal relationships that could have appeared to influence the work reported in this paper.

## Data Availability

All data used in this study will be made accessible and open immediately upon publication.

## References

[bib1] Begus K., Lloyd-Fox S., Halliday D., Papademetriou M., Darboe M.K., Prentice A.M., Elwell C.E. (2016). Oxygen transport to tissue XXXVII.

[bib2] Benjamini Y., Hochberg Y. (1995). Controlling the false discovery rate: a practical and powerful approach to multiple testing. J. R. Stat. Soc. Ser. B Methodol..

[bib3] Berens A.E., Kumar S., Tofail F., Jensen S.K., Alam M., Haque R., Nelson C.A. (2019). Cumulative psychosocial risk and early child development: validation and use of the Childhood Psychosocial Adversity Scale in global health research. Pediatr. Res..

[bib4] Bick J., Nelson C.A. (2016). Early adverse experiences and the developing brain. Neuropsychopharmacology.

[bib5] Blasi A., Lloyd-Fox S., Katus L., Elwell C.E. (2019).

[bib6] Campbell S.B., Brownell C.A., Hungerford A., Spieker S.J., Mohan R., Blessing J.S. (2004). The course of maternal depressive symptoms and maternal sensitivity as predictors ofattachment security at 36 months. Dev. Psychopathol..

[bib7] Campbell S.B., Matestic P., von Stauffenberg C.V., Mohan R., Kirch- ner (2007). Trajectories of maternal depressive symptoms, maternal sensitivity, and children’s functioning at school entry. Dev. Psychol..

[bib8] Choe D.E., Olson S.L., Sameroff A.J. (2013). Effects of early maternal distress and parenting on the development of children’s self-regulation and externalizing behavior. Dev. Psychopathol..

[bib9] Choi J., Jeong B., Rohan M.L., Polcari A.M., Teicher M.H. (2009). Preliminary evidence for white matter tract abnormalities in young adults exposed to parental verbal abuse. Biol. Psychiatry.

[bib10] Chowdhury, S.C., Razzaque, M.M., Helali, M.M., & Bodén, H. (2010). Assessment of noise pollution in Dhaka city. In 17th International Congress on Sound and Vibration, Cairo, Egypt, 2010–07-18–2010-07–22.

[bib11] Cummings E.M., Davies P.T. (2010).

[bib12] Cunradi C.B., Caetano R., Clark C., Schafer J. (2000). Neighborhood poverty as a predictor of intimate partner violence among White, Black, and Hispanic couples in the United States: a multilevel analysis. Ann. Epidemiol..

[bib13] Cutting A.L., Dunn J. (1999). Theory of mind, emotion understanding, language, and family background: Individual differences and interrelations. Child Dev..

[bib14] Davies P.T., Sturge-Apple M.L., Cicchetti D., Cummings E.M. (2007). The role of child adrenocortical functioning in pathways between interparental conflict and child maladjustment. Dev. Psychol..

[bib15] Di Lorenzo R.*, Pirazzoli L.*, Blasi A., Bulgarelli C., Hakuno Y., Minagawa Y., Brigadoi S. (2019). Recommendations for motion correction of infant fNIRS data applicable to multiple data sets and acquisition systems. NeuroImage.

[bib16] Duncan G.J., Brooks‐Gunn J., Klebanov P.K. (1994). Economic deprivation and early childhood development. Child Dev..

[bib17] Dunn J., Brown J., Slomkowski C., Tesla C., Youngblade L. (1991). Young children’s understanding of other people’s feelings and beliefs: Individual differences and their antecedents. Child Dev..

[bib18] Elder G., Baltes P., Brim O. (1979).

[bib19] Ellis B.J., Abrams L.S., Masten A.S., Sternberg R.J., Tottenham N., Frankenhuis W.E. (2020). Hidden talents in harsh environments. Dev. Psychopathol..

[bib20] Evans G.W., English K. (2002). The environment of poverty: multiple stressor exposure, psychophysiological stress, and socioemotional adjustment. Child Dev..

[bib21] Evans G.W., Kim P. (2010). Multiple risk exposure as a potential explanatory mechanism for the socioeconomic status–health gradient. Ann. N. Y. Acad. Sci..

[bib22] Everdell N.L., Gibson A.P., Tullis I.D.C., Vaithianathan T., Hebden J.C., Delpy D.T. (2005). A frequency multiplexed near-infrared topography system for imaging functional activation in the brain. Rev. Sci. Instrum..

[bib23] Farah M.J. (2017). The neuroscience of socioeconomic status: correlates, causes, and consequences. Neuron 96. 1.

[bib24] Fu X., Richards J.E. (2021). Investigating developmental changes in scalp-to-cortex correspondence using diffuse optical tomography sensitivity in infancy. Neurophotonics.

[bib25] Graham A.M., Fisher P.A., Pfeifer J.H. (2013). What sleeping babies hear: a functional MRI study of interparental conflict and infants’ emotion processing. Psychol. Sci..

[bib26] Grossmann T. (2015). The development of social brain functions in infancy. Psychol. Bull..

[bib27] Guajardo N.R., Snyder G., Petersen R. (2009). Relationships among parenting practices, parental stress, child behaviour, and children’s social‐cognitive development. Infant Child Dev. Int. J. Res. Pract..

[bib28] Hernandez, S.M., & Pollonini, L. (2020). NIRSplot: a tool for quality assessment of fNIRS scans. In Optics and the Brain (pp. BM2C-5). Optical Society of America.

[bib29] Huppert T.J., Diamond S.G., Franceschini M.A., Boas D.A. (2009). HomER: a review of time-series analysis methods for near-infrared spectroscopy of the brain. Appl. Opt..

[bib30] Jensen S.K., Xie W., Kumar S., Haque R., Petri W.A., Nelson C.A. (2021). Associations of socioeconomic and other environmental factors with early brain development in Bangladeshi infants and children. Dev. Cogn. Neurosci..

[bib31] Jensen S.K.G., Dumontheil I., Barker E.D. (2014). Developmental inter‐relations between early maternal depression, contextual risks, and interpersonal stress, and their effect on later child cognitive functioning. Depress Anxiety.

[bib32] Katus L., Mason L., Milosavljevic B., McCann S., Rozhko M., Moore S.E., Prentice A. (2020). ERP markers are associated with neurodevelopmental outcomes in 1–5 month old infants in rural Africa and the UK. NeuroImage.

[bib33] Kraus M.W., Piff P.K., Mendoza-Denton R., Rheinschmidt M.L., Keltner D. (2012). Social class, solipsism, and contextualism: how the rich are different from the poor. Psychol. Rev..

[bib34] Krishnakumar A., Buehler C. (2000). Interparental conflict and parenting behaviors: a meta‐analytic review. Fam. Relat..

[bib35] Lloyd-Fox S., Blasi A., Elwell C.E. (2010). Illuminating the developing brain: the past, present and future of functional near infrared spectroscopy. Neurosci. Biobehav. Rev..

[bib36] Lloyd-Fox S., Richards J.E., Blasi A., Murphy D.G., Elwell C.E., Johnson M.H. (2014). Coregistering functional near-infrared spectroscopy with underlying cortical areas in infants. Neurophotonics.

[bib37] Lloyd-Fox S., Begus K., Halliday D., Pirazzoli L., Blasi A., Papademetriou M., Elwell C.E. (2017). Cortical specialisation to social stimuli from the first days to the second year of life: a rural Gambian cohort. Dev. Cogn. Neurosci..

[bib38] Lloyd‐Fox S., Blasi A., Volein A., Everdell N., Elwell C.E., Johnson M.H. (2009). Social perception in infancy: A near infrared spec‐ troscopy study. Child Dev..

[bib39] Lloyd‐Fox S., Blasi A., McCann S., Rozhko M., Katus L., Mason L., Austin T., Moore S.E., Elwell C.E., Bright Project Team (2019). Habituation and novelty detection fNIRS brain responses in 5‐and 8‐month‐old infants: The Gambia and UK. Developmental Science.

[bib40] Lloyd‐Fox S., Blasi A., Mercure E., Elwell C.E., Johnson M.H. (2012). The emergence of cerebral specialization for the human voice over the first months of life. Soc. Neurosci..

[bib41] Lloyd‐Fox S., Blasi A., Elwell C.E., Charman T., Murphy D., Johnson M.H. (2013). Reduced neural sensitivity to social stimuli in infants at risk for autism. Proc. R. Soc. B: Biol. Sci..

[bib42] Lloyd‐Fox S., Papademetriou M., Darboe M.K., Everdell N.L., Wegmuller R., Prentice A.M., Elwell C.E. (2014). Functional near infrared spectroscopy (fNIRS) to assess cognitive function in infants in rural Africa. Sci. Rep..

[bib43] Lloyd‐Fox S., Blasi A., Pasco G., Gliga T., Jones E.J.H., Murphy D.G.M., Yemane F. (2018). Cortical responses before 6 months of life associate with later autism. Eur. J. Neurosci..

[bib44] Lovejoy C.M., Graczyk P.A., O’Hare E., Neuman G. (2000). Maternal depression and parenting behavior. Clin. Psychol. Rev..

[bib45] Lund C., Breen A., Flisher A.J., Kakuma R., Corrigall J., Joska J.A., Patel V. (2010). Poverty and common mental disorders in low and middle income countries: A systematic review. Soc. Sci. Med..

[bib46] McCoy D.C., Peet E.D., Ezzati M., Danaei G., Black M.M., Sudfeld C.R., Fink G. (2016). Early childhood developmental status in low-and middle-income countries: national, regional, and global prevalence estimates using predictive modeling. PLoS Med..

[bib47] McLaughlin K.A., Sheridan M.A., Lambert H.K. (2014). Childhood adversity and neural development: deprivation and threat as distinct dimensions of early experience. Neurosci. Biobehav. Rev..

[bib48] McLoyd V.C. (1990). The impact of economic hardship on Black families and children: Psychological distress, parenting, and socioemotional development. Child Dev..

[bib49] McLoyd V.C. (1997). The impact of poverty and low socioeconomic status on the socioemotional functioning of African-American children and adolescents: Mediating effects. Soc. Emot. Adjust. Fam. Relat. Ethn. Minor. Fam..

[bib50] McLoyd V.C. (1998). Socioeconomic disadvantage and child development. Am. Psychol..

[bib51] Molavi B., Dumont G.A. (2012). Wavelet-based motion artifact removal for functional near-infrared spectroscopy. Physiol. Meas..

[bib52] Pears K.C., Moses L.J. (2003). Demographics, parenting, and theory of mind in preschool children. Soc. Dev..

[bib53] Perdue K.L., Jensen S.K., Kumar S., Richards J.E., Kakon S.H., Haque R., Nelson C.A. (2019). Using functional near‐infrared spectroscopy to assess social information processing in poor urban Bangladeshi infants and toddlers. Dev. Sci..

[bib54] Perra O., Phillips R., Fyfield R., Waters C., Hay D.F. (2015). Does mothers’ postnatal depression influence the development of imitation?. J. Child Psychol. Psychiatry.

[bib55] Richards J.E., Sanchez C., Phillips-Meek M., Xie W. (2016). A database of age-appropriate average MRI templates. Neuroimage.

[bib56] Ridley M., Rao G., Schilbach F., Patel V. (2020). Poverty, depression, and anxiety: causal evidence and mechanisms. Science.

[bib57] Rohrer L.M., Cicchetti D., Rogosch F.A., Toth S.L., Maughan A. (2011). Effects of maternal negativity and of early and recent recurrent depressive disorder on children’s false belief understanding. Dev. Psychol..

[bib58] Ruffman T., Perner J., Parkin L. (1999). How parenting style affects false belief understanding. Soc. Dev..

[bib59] Sanchez C.E., Richards J.E., Almli C.R. (2012). Age-specific MRI templates for pediatric neuroimaging. Dev. Neuropsychol..

[bib60] Sareen J., Afifi T.O., McMillan K.A., Asmundson G.J. (2011). Relationship between household income and mental disorders: findings from a population-based longitudinal study. Arch. Gen. Psychiatry.

[bib61] Scholkmann F., Spichtig S., Muehlemann T., Wolf M. (2010). How to detect and reduce movement artifacts in near-infrared imaging using moving standard deviation and spline interpolation. Physiol. Meas..

[bib62] Tang Y., Hojatkashani C., Dinov I.D., Sun B., Fan L., Lin X., Toga A.W. (2010). The construction of a Chinese MRI brain atlas: a morphometric comparison study between Chinese and Caucasian cohorts. Neuroimage.

[bib63] Tang Y., Zhao L., Lou Y., Shi Y., Fang R., Lin X., Toga A. (2018). Brain structure differences between C hinese and C aucasian cohorts: A comprehensive morphometry study. Hum. brain Mapp..

[bib64] Tomalski P., Moore D.G., Ribeiro H., Axelsson E.L., Murphy E., Karmiloff‐Smith A., Kushnerenko E. (2013). Socioeconomic status and functional brain development–associations in early infancy. Dev. Sci..

[bib65] Tomoda A., Sheu Y.S., Rabi K., Suzuki H., Navalta C.P., Polcari A., Teicher M.H. (2011). Exposure to parental verbal abuse is associated with increased gray matter volume in superior temporal gyrus. Neuroimage.

[bib66] Wade M., Moore C., Astington J.W., Frampton K., Jenkins J.M. (2015). Cumulative contextual risk, maternal responsivity, and social cognition at 18 months. Dev. Psychopathol..

[bib67] Wijeakumar S., Kumar A., Delgado Reyes L.M., Tiwari M., Spencer J.P. (2019). Early adversity in rural India impacts the brain networks underlying visual working memory. Dev. Sci..

[bib68] Winer A.C., Thompson R. (2011). How poverty and depression impact a child ’s social and emotional competence. Policy Brief Cent. Poverty Res..

[bib69] Xie W., Richards J.E., Lei D., Zhu H., Lee K., Gong Q. (2015). The construction of MRI brain/head templates for Chinese children from 7 to 16 years of age. Dev. Cogn. Neurosci..

[bib70] Xie W., Jensen S.K., Wade M., Kumar S., Westerlund A., Kakon S.H., Nelson C.A. (2019). Growth faltering is associated with altered brain functional connectivity and cognitive outcomes in urban Bangladeshi children exposed to early adversity. BMC Med..

[bib71] Xie W., Kumar S., Kakon S.H., Haque R., Petri W.A., Nelson C.A. (2019). Chronic inflammation is associated with neural responses to faces in Bangladeshi children. Neuroimage.

