# Supplementary material for: Association of psychosocial adversity and social information processing in children raised in a low-resource setting: an fNIRS study
Source: Dev Cogn Neurosci. 2022 Jun 18;56:101125. doi: 10.1016/j.dcn.2022.101125 (PMC9241055; doi:10.1016/j.dcn.2022.101125)
Supplement: Supplementary file 1 — Supplementary material [file mmc1.docx]

**Supplementary material:**

Figure S1 Grand averaged time courses of the response to the social visual stimulus in each channel at 6 months. Red is HbO_2_, blue is HHb and green is total haemoglobin. X axis is time in seconds, Y axis is concentration expressed in μM. Next to each row of channels, channels highlighted in blue show the location of those channels on a head model.

Figure S2 Grand averaged time courses of the response to the social visual stimulus in each channel at 24 months. Red is HbO_2_, blue is HHb and green is total haemoglobin. X axis is time in seconds, Y axis is concentration expressed in μM. Next to each row of channels, channels highlighted in blue show the location of those channels on a head model.

Figure S3 Grand averaged time courses of the response to the social visual stimulus in each channel at 36 months. Red is HbO_2_, blue is Hbb and green is total haemoglobin. X axis is time in seconds, Y axis is concentration expressed in μM. Next to each row of channels, channels highlighted in blue show the location of those channels on a head model.

Figure S4 Channel locations along with color and size of dots indicating what percentage of participants had that channel included in a ROI. Data shown for each age group and ROI

Figure S5 Grand averaged haemodynamic HbO_2_ (left) and HHb (right) time courses averaged across conditions (VS, AS, AN) at 6mo (top panel), 24mo (middle panel) and 36mo (bottom panel). The red and blue lines show the response per channel; and the dashed grey lines mark the start and end of the time window chosen for the statistical analyses

Figure S6 Comparison of channel-by-channel and ROI analyses at 6mo.Top panels: a schematic of the infant head showing channels with statistically significant HbO2 responses (p<.005) for visual social contrast (orange) and the auditory social contrast (green). Bottom panels: ROIs with statistically significant HbO_2_ responses for visual social contrast (orange) and the auditory social contrast (green). If a channel or an ROI are circled in dark blue, it indicates that it has survived FDR corrections. Blue boxes indicate channels rejected due to cross talk issues.

Figure S7 Comparison of channel-by-channel and ROI analyses at 6mo.Top panels: a schematic of the infant head showing channels with statistically significant HbO2 responses (p<.005) for visual social contrast (orange) and the auditory social contrast (green). Bottom panels: ROIs with statistically significant HbO_2_ responses for visual social contrast (orange) and the auditory social contrast (green). If a channel or an ROI are circled in dark blue, it indicates that it has survived FDR corrections. Blue boxes indicate channels rejected due to cross talk issues.

Figure S8 Comparison of channel-by-channel and ROI analyses at 6mo.Top panels: a schematic of the infant head showing channels with statistically significant HbO2 responses (p<.005) for visual social contrast (orange) and the auditory social contrast (green). Bottom panels: ROIs with statistically significant HbO_2_ responses for visual social contrast (orange) and the auditory social contrast (green). If a channel or an ROI are circled in dark blue, it indicates that it has survived FDR corrections. Blue boxes indicate channels rejected due to cross talk issues.

Table S1 Average number of channels included in each ROI (number in brackets indicates SD).

|  | **IFG** | | **aMTG-STG** | | **pMTG-STG/TPJ** | |
| --- | --- | --- | --- | --- | --- | --- |
|  | *r* | *l* | *r* | *l* | *r* | *l* |
| 6mo | 2.5 (1.2) | 3 (1.1) | 2 (2.1) | 1.7 (1.8) | 7 (1.7) | 6.8 (1.59) |
| 24mo | 3 (1.2) | 3 (1.2) | 0.5 (0.9) | 0.7 (1.1) | 7.6 (1.5) | 7.5 (1.5) |
| 36mo | 3.8 (1.6) | 2.15 (1.2) | 1.56 (1.31) | 1.6 (1.4) | 6.3 (1.9) | 7.9 (1.5) |

**Comparing different regions and age groups**

To compare responses in different brain regions and age groups, we employed a relative measure: percentage of participants with significant response in each ROI.

To calculate these percentages, for each individual we calculated whether they had a significant response to the visual social trails vs. baseline (one sample t-tests of the response to each social visual trial (ΔHbO2 averaged between 12 and 16 seconds post stimulus onset) versus 0) and a significant difference between the responses to the social and non-social auditory trials (paired sample t-tests).

We used Chi-Square tests to compare both within age group, to look for effects of ROI and hemisphere, and within ROI to test effects of age.

Overall, we found no effect of ROI and hemisphere within each age group. Specifically:

At 6 months % of participants with significant responses in the visual social contrast does not differ across ROIs and hemispheres X2(5, 823)=2.308, p=.805 and % of participants with significant responses in the auditory social contrast does not differ across ROIs and hemispheres X2(5, 772)=2.611, p=.760.

At 24 months % of participants with significant responses in the visual social contrast does not differ across ROIs and hemispheres X2(5, 645)=8.387, p=.136 and % of participants with significant responses in the auditory social contrast does not differ across ROIs and hemispheres X2(5, 591)=2.812, p=.729.

At 36 months % of participants with significant responses in the visual social contrast does not differ across ROIs and hemispheres X2(5, 1032)=9.338, p=.096 and % of participants with significant responses in the auditory social contrast does not differ across ROIs and hemispheres X2(5, 910)=1.222, p=.943.

To look at age effects we compared % of participants with significant responses in each ROI across different ages. Overall, we found no effect of age within each ROI. Specifically:

For IFG, we found no effect of age either for the visual social contrast X2(5, 953)=4.152, p=.528 or for the auditory social contrast X2(5, 887)=1.773, p=.880.

For aMTG-STG, we found no effect of age either for the visual social contrast X2(5, 548)=.734, p=.981 or for the auditory social contrast X2(5, 517)=4.165, p=.526.

For pMTG-STG/TPJ, we found no effect of age either for the visual social contrast X2(5, 999)=7.097, p=.214 or for the auditory social contrast X2(5, 869)=1.274, p=.938.

The table below shows percentages of participants with significant responses for both visual and auditory contrasts and summarized findings from Chi-square tests reported above.
